# Split-wrmScarlet and split-sfGFP: tools for faster, easier fluorescent labeling of endogenous proteins in *Caenorhabditis elegans*

**DOI:** 10.1093/genetics/iyab014

**Published:** 2021-02-02

**Authors:** Jérôme Goudeau, Catherine S Sharp, Jonathan Paw, Laura Savy, Manuel D Leonetti, Andrew G York, Dustin L Updike, Cynthia Kenyon, Maria Ingaramo

**Affiliations:** 1 Calico Life Sciences LLC, South San Francisco, CA 94080, USA; 2 Mount Desert Island Biological Laboratory, Bar Harbor, ME 04672, USA; 3 Chan Zuckerberg Biohub, San Francisco, CA 94158, USA

**Keywords:** *C. elegans*, Cas9, CRISPR, genome engineering, GFP, protein localization, mScarlet, germline, wrmScarlet

## Abstract

We create and share a new red fluorophore, along with a set of strains, reagents and protocols, to make it faster and easier to label endogenous *Caenorhabditis elegans* proteins with fluorescent tags. CRISPR-mediated fluorescent labeling of *C. elegans* proteins is an invaluable tool, but it is much more difficult to insert fluorophore-size DNA segments than it is to make small gene edits. In principle, high-affinity asymmetrically split fluorescent proteins solve this problem in *C. elegans*: the small fragment can quickly and easily be fused to almost any protein of interest, and can be detected wherever the large fragment is expressed and complemented. However, there is currently only one available strain stably expressing the large fragment of a split fluorescent protein, restricting this solution to a single tissue (the germline) in the highly autofluorescent green channel. No available *C. elegans* lines express unbound large fragments of split red fluorescent proteins, and even state-of-the-art split red fluorescent proteins are dim compared to the canonical split-sfGFP protein. In this study, we engineer a bright, high-affinity new split red fluorophore, split-wrmScarlet. We generate transgenic *C. elegans* lines to allow easy single-color labeling in muscle or germline cells and dual-color labeling in somatic cells. We also describe a novel expression strategy for the germline, where traditional expression strategies struggle. We validate these strains by targeting split-wrmScarlet to several genes whose products label distinct organelles, and we provide a protocol for easy, cloning-free CRISPR/Cas9 editing. As the collection of split-FP strains for labeling in different tissues or organelles expands, we will post updates at doi.org/10.5281/zenodo.3993663

## Introduction

Genetically expressed fluorophores are essential tools for visualizing and quantifying cellular proteins. In *Caenorhabditis elegans*, fluorescent proteins have traditionally been introduced on extrachromosomal arrays (Kimble *et al.* 1982; Mello et al. 1991) or via MosSCI-based integration (Frøkjær-Jensen *et al.* 2008, 2012). These methods have enabled important discoveries but can also lead to artifacts due to supraphysiological gene-expression levels and lack of endogenous regulatory control. In recent years, the repertoire of *C. elegans* transgenic tools has expanded (see Nance and Frøkjær-Jensen 2019, for review), particularly due to advances in CRISPR/Cas9 genome-editing technologies (Paix *et al.* 2014; Dickinson and Goldstein 2016). CRISPR/Cas9 allows precise transgene insertion by homology-directed repair (HDR) and can be used to label an endogenous gene at its native locus with a fluorescent protein (Friedland *et al.* 2013; Dokshin *et al.* 2018; Farboud *et al.* 2019; Vicencio *et al.* 2019).

However, relative to CRISPR/Cas9-mediated integration of smaller transgenes, genomic insertion of large DNA fragments like those encoding fluorescent proteins remains a challenge, both because repair with double-stranded templates is less efficient than repair with single-stranded oligodeoxynucleotide donors (ssODN) (Farboud *et al.* 2019), and because of the requirement for cloning to prepare the HDR donor template. Recent methods such as “hybrid” (Dokshin *et al.* 2018) and “nested” (Vicencio *et al.* 2019) CRISPR remove the need for cloning but still require preparation of the DNA template or several rounds of injections and selection of transgenic progeny. As a result, using CRISPR with small ssODN templates is currently faster, easier, cheaper, and more efficient than with large templates. In our lab, we routinely make *C. elegans* genome edits with short ssODNs with almost guaranteed success. In contrast, in our experience, large edits using double-stranded DNA templates have higher failure rates and are more time-consuming.

**Figure 1:**
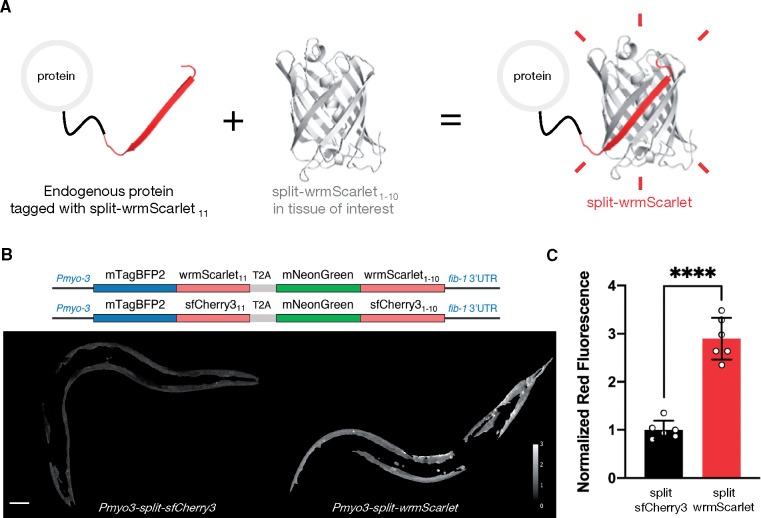
Engineering and evaluating split-wrmScarlet. (A) Principle of endogenous protein labeling with split-wrmScarlet. The protein structure from split-wrmScarlet was generated using Phyre2 and PyMOL. (B) Schematic of the plasmids encoding split-wrmScarlet and split-sfCherry3. Each plasmid consists of the large FP_1–10_ sequence fused to mNeonGreen, and the corresponding small FP_11_ sequence fused to mTagBFP2. The T2A sequence ensures that mTagBFP2::FP_11_ and the corresponding mNeonGreen::FP_1–10_ are separated. The images are representative displays of the ratio of red to green fluorescence intensity from images acquired under identical conditions after background subtraction and masking with the same threshold. Scale bar, 50 µm. (C) Emission intensities from split-sfCherry3 and split-wrmScarlet normalized to mNeonGreen. Mean ± SD. Circles are individuals (*n* = 6 for each split fluorescent protein). ****P < 0.0001.

Our preferred approach is to combine the utility of full-length fluorescent proteins with the convenience of short genomic edits, by using high-affinity asymmetrically split fluorescent proteins (Cabantous *et al.* 2005). These fluorophores typically separate a GFP-like protein between the 10th and 11th strands of the beta barrel, splitting it asymmetrically into a large (FP_1–10_) and a small (FP_11_) fragment. The fragments are not individually fluorescent, but upon binding one another, recapitulate the fluorescent properties of an intact fluorophore (Figure 1A). Unlike the low-affinity split fluorescent proteins used in BiFC assays (Hu *et al.* 2002), high-affinity binding between the fragments is critical here. Our preferred approach for tagging a new cellular protein begins with a *C. elegans* strain expressing the large FP_1–10_ fragment in cells of interest, unattached to any cellular protein. This way, only the small FP_11_ fragment (<72 nt) needs to be inserted to tag the target protein, which will only fluoresce in compartments where it can bind the large fragment. These short insertions tend to be faster, easier, and more reliable than inserting a > 600 nt full-length fluorescent protein (Paix *et al*. 2015; Prior *et al.* 2017, Dokshin *et al.* 2018; Richardson *et al.* 2018). Therefore, collections of *C. elegans* lines stably expressing the large FP_1–10_ in different tissues are an invaluable resource allowing rapid fluorescent tagging in a cell type of choice. Stable lines with red FP_1–10_ fragments would be especially useful, given *C. elegans*’ substantial autofluorescence in the GFP channel.

Green and red asymmetrically split fluorescent proteins have been used to combine cell and protein specificity in *C. elegans* neurons and synapses (Noma *et al.* 2017; Feng *et al.* 2019; He *et al.* 2019); however, these strains used extrachromosomal arrays, not stable lines, which are more time-consuming to maintain and can have variable expression levels. To the best of our knowledge, there is only one available unbound FP_1–10_ stable *C. elegans* line, which expresses sfGFP_1–10_ in the germline (Hefel and Smolikove 2019), and there are no available lines with red FP_1–10_ fragments. Existing red split fluorophores are also much dimmer in *C. elegans* than green ones, despite recent improvements like split-sfCherry3 (Feng *et al.* 2019). In addition, we often struggle to express genome-integrated full-length fluorescent protein fusions in the germline, potentially due to generational silencing.

Here, we describe tools that reduce these obstacles for convenient fluorescent labeling of endogenous *C. elegans* proteins. We engineer split-wrmScarlet, a new split red fluorescent protein based on mScarlet (Bindels *et al.* 2017; El Mouridi *et al.* 2017), which is three times brighter in worms than split-sfCherry3 (https://www.addgene.org/138966). We generate and share *C. elegans* lines carrying single-copy insertions of split-wrmScarlet_1–10_ expressed broadly in somatic cells (https://cgc.umn.edu/strain/CF4582), and specifically in muscle (https://cgc.umn.edu/strain/CF4610). We also describe a novel approach to make *C. elegans* lines with robust germline expression of exogenous proteins that appears to be resistant to generational silencing. We use this approach to make a germline-specific split-wrmScarlet_1–10_ strain (https://cgc.umn.edu/strain/DUP237).

We provide a protocol for an easy, cloning-free method to label endogenous genes with FP_11_s using CRISPR/Cas9, commercially available synthetic ssODN, and microinjection (dx.doi.org/10.17504/protocols.io.bamkic4w). We validate this protocol by targeting split-wrmScarlet_11_ to six different genes whose products have distinct cellular locations. We show that labeling with tandem split-wrmScarlet_11_-repeats increases fluorescence *in vivo*, and we provide the plasmid necessary to generate the dsDNA template (https://www.addgene.org/158533). We also generate a strain expressing an integrated copy of sfGFP_1–10_ (Pédelacq *et al.* 2006) broadly in somatic cells (https://cgc.umn.edu/strain/CF4587), and a strain expressing sGFP2_1–10_ (Köker *et al.* 2018) specifically in the germline (https://cgc.umn.edu/strain/DUP223). Finally, we generate a dual-color strain expressing both sfGFP_1–10_ and split-wrmScarlet_1–10_ in somatic cells (https://cgc.umn.edu/strain/CF4588), for two-color applications such as colocalization studies or organelle interaction. We hope that these resources will facilitate the study of *C. elegans* biology. As the collection of split-FP strains and related resources for labeling different tissues, organelles and proteins expands, we will post updates at doi.org/10.5281/zenodo.3993663.

## Materials and methods

### Mutagenesis and screening

For the initial screenings in *Escherichia coli*, we introduced a 32 amino-acid spacer between the 10th and 11th β-strands of full-length mScarlet in a pRSET vector (Feng *et al.* 2017). This starting construct was nonfluorescent, but we restored low fluorescence levels by introducing the superfolder mutation G220A. Semi-random mutagenesis was carried out using rolling-circle amplification with NNK primers at positions I8, K10, F15, G32, Q43, A45, K46, L47, G52, G53, D60, S63, P64, Q65, F66, S70, R71, T74, K75, D79, Y84, W94, R96, T107, V108, Q110, E115, L125, R126, T128, K139, K140, W144, E145, S147, T148, E149, R150, I162, K163, M164, L175, F178, K179, K183, K185, K186, N195, R198, I202, T203, S204, D208, Y209, T210, V211, V212, E213, Q214, Y215, E216, R217, S218, E219, A220, H222, S223, T224, G225, G226, M227, D228, and E229 with Phusion polymerase (NEB) in GC buffer, followed by pooling of the PCR products, *DpnI* digestion and transformation into BL21(DE3) *E. coli*. These positions covered areas deemed important for brightness or stability, and the interface between FP_11_ and FP_1–10_. Primers were resynthesized if a mutation interfered with neighboring mutagenic primer binding. The brightest three to five colonies were identified using a Leica M165 FC fluorescent stereomicroscope, and their plasmid DNA subjected to a new mutagenesis round. After five rounds, we separated the two fragments of a version of split-wrmScarlet (which had fluorescence comparable to mScarlet) into two *S. cerevisiae* plasmids to test for complementation. Because we did not detect fluorescence, we continued selection using two plasmids in yeast. For screening on two plasmids, a pRSET vector expressing split-wrmScarlet_1–10_ and a pD881-MR vector (ATUM) expressing mTagBFP-split-wrmScarlet_11_ (without the MDELYK tail from the C-terminus) were used to perform the semi-random mutagenesis. The libraries were co-electroporated into *E. coli* and expression was induced with 1% rhamnose and 1 mM IPTG. The library was enriched for fluorescent clones using FACS, and then subcloned to make pRS-GPD-split-wrmScarlet_1–10_ and p416-TEF-membrane-mTagBFP-split-wrmScarlet_11_. The yeast plasmids were co-transformed into a URA^-^, HIS^-^, LEU^-^, MET^-^*S. cerevisiae* strain and selected for in SC media without uracil and histidine, and FACS was used again for enrichment of clones with the highest red to blue ratio. After three rounds of semi-random mutagenesis with the two-plasmid strategy, a final round of random mutagenesis was performed using the GeneMorph II kit (Agilent). Yeast plasmids are available through Addgene (https://www.addgene.org/158585/, https://www.addgene.org/158584/), and *E. coli* plasmid sequences are present in Supplementary Table S5.

### *C. elegans* strains and maintenance

Animals were cultured under standard growth conditions with *E. coli* OP50 at 20°C (Brenner 1974). Strains generated in this work are listed in the Supplementary Table S3.

### Nucleic acid reagents

Synthetic nucleic acids were purchased from Integrated DNA Technologies (IDT), GenScript or Genewiz. For knock-in of a single split-wrmScarlet_11_ or sfGFP_11_ sequence, 200-mer HDR templates were ordered in ssODN form (synthetic ssODN) from IDT. For knock-in of split-wrmScarlet_11_ repeats, HDR templates were ordered in dsDNA form (plasmids) from GenScript or Genewiz. For plasmids injected as extrachromosomal arrays, sequences were synthesized and cloned into the pUC57 vector (Genewiz). The complete set of crRNAs and DNA sequences and plasmids used for the experiments described here can be found in Supplementary Tables S1, S4, and S5.

### Strain generation: CRISPR/Cas9-triggered homologous recombination

CRISPR insertions were performed using published protocols (Paix *et al*. 2015; Paix* et al.* 2016). Ribonucleoprotein complexes (protein Cas9, tracrRNA, and crRNA) and DNA templates were microinjected into the gonad of young adults using standard methods (Evans 2006). Injected worms were singled and placed at 25°C overnight. All crRNA and DNA template sequences used to generate the strains described in this work are listed in the Supplementary Table S4. Split-wrmScarlet_11_ and sfGFP_11_ integrants were identified by screening for fluorescence in the F_1_ or F_2_ progeny of injected worms. The co-CRISPR *dpy-10(cn64)* mutation was used as a marker when generating nonfluorescent strains. The CF4582 strain *muIs252[Peft-3::split-wrmScarlet_1–10_::unc-54 3'UTR Cbr-unc-119(+)] II; unc-119(ed3) III* was generated by replacing the *tir-1::mRuby* sequence from the strain CA1200 *ieSi57 II; unc-119(ed3) III* (Zhang *et al.* 2015) with the *split-wrmScarlet_1–10_* sequence. The CF4587 strain *muIs253[Peft-3::sfGFP_1_*_–_*_10_::unc-54 3'UTR Cbr-unc-119(+)] II; unc-119(ed3) III* was generated by replacing the *let-858* promoter from the strain COP1795 *knuSi785 [pNU1687(Plet-858::sfGFP_1–10_::unc-54 3’UTR unc-119(+))] II; unc-119(ed3) III* with the *eft-3* (also known as *eef-1A.1*) promoter. Both CF4582 and CF4587 strains were generated using long, partially single-stranded DNA donors (Dokshin *et al.* 2018). The CF4610 strain *muIs257[Pmyo-3::split-wrmScarlet_1–10_::unc-54 3'UTR] I* was generated by inserting the split-wrmScarlet_1–10_ sequence in the WBM1126 strain following the SKI LODGE protocol (Silva-García *et al.* 2019). The strains PHX731 *vha-13(syb731[wrmScarlet::vha-13]) V* and PHX1049 *vha-13(syb1049[gfp::vha-13]) V* were generated by SunyBiotech’s CRISPR services. Strains generated were genotyped by Sanger sequencing of purified PCR products (Genewiz).

### Strain generation: Mos1-mediated single-copy insertion

The COP1795 strain was generated by NemaMetrix’s MosSCI services. The PHX1797 strain was generated by SunyBiotech’s MosSCI services, using a codon-optimized sequence of split-wrmScarlet_1–10_ with three introns, and engineered to avoid piRNA recognition transgene silencing (Wu *et al.* 2018; Zhang *et al.* 2018; Supplementary Table S1).

### Strain generation: genetic crosses

The following *C. elegans* strains were created by standard genetic crosses: CF4588 *muIs253[Peft-3::sfGFP_1–10_::unc-54 3'UTR Cbr-unc-119(+)] muIs252[Peft-3::split-wrmScarlet_1–10_::unc-54 3'UTR Cbr-unc-119(+)] II; unc-119(ed3) III* and CF4602 *muIs253[Peft-3::sfGFP_1–10_::unc-54 3'UTR Cbr-unc-119(+)] muIs252[Peft-3::split-wrmScarlet_1–10_::unc-54 3'UTR Cbr-unc-119(+)] II; unc-119(ed3) III; fib-1(muIs254[split-wrmScarlet_11_::fib-1]) his-3(muIs255[his-3::sfGFP_11_]) V*. Nonfluorescent parental lines CF4582, CF4587 and CF4610 generated using *dpy-10(cn64)* co-CRISPR were backcrossed at least once.

### Strain generation: plasmid microinjection

*Peft-3::3NLS::mTagBFP2::split-wrmScarlet_11_::T2A::mNeonGreen::split-wrmScarlet_1–10_::fib-1 3’UTR*, *Peft-3::3NLS::mTagBFP2::sfCherry3_11_::T2A::mNeonGreen::sfCherry3_1–10_::fib-1 3’UTR*, *Pmyo-3::mTagBFP2::split-wrmScarlet_11_::T2A::mNeonGreen::split-wrmScarlet_1–10_::fib-1 3’UTR*, or *Pmyo-3::mTagBFP2::sfCherry3_11_::T2A::mNeonGreen::sfCherry3_1–10_::fib-1 3’UTR* constructs were microinjected at (20 ng/μL) using a standard microinjection procedure (Mello et al. 1991). Germline gene expression was achieved using a microinjection-based protocol with diluted transgenic DNA (Kelly *et al.* 1997), *Psun-1::mNeonGreen::linker::split-wrmScarlet_11_::tbb-2 3’UTR* construct (5 ng/µL) was co-injected with PvuII-digested genomic DNA fragments from *E. coli* (100 ng/µL). Plasmid sequences are listed in Supplementary Table S5.

### Germline strain generation: *glh-1::T2A::split-wrmScarlet_1–10_ and glh-1::T2A::sGFP2_1_*_–__*10*_

Using CRISPR/Cas9, the C-terminus of *glh-1* was tagged with either T2A::split-wrmScarlet_1-11_ or T2A::sGFP2_1-11,_ a split superfolder GFP variant optimized for brightness and photostability (Köker *et al.* 2018). Fluorescence originating from these full-length fusions was present throughout the cytoplasm and nuclei of adult germ cells and gametes, with the maternally deposited signal persisting through the early stages of embryogenesis and larval development (Supplementary Figure S8, A and B, top panels). After verifying fluorescence, we used a precise CRISPR/Cas9 deletion of either split-wrmScarlet_11_ or sGFP2_11_ to convert these FP_1–11_ strains into FP_1–10_ strains, DUP237 *glh-1(sam140[glh-1::T2A::split-wrmScarlet_1_*_–_*_10_]) I* and DUP223 *glh-1(sam129[glh-1::T2A::sGFP2_1_*_–_*_10_]) I* and corroborated the absence of fluorescence (Supplementary Figure S8, A and B, middle panels). The crRNAs, ssDNAs and dsDNA template sequences are described in Supplementary Tables S1–S4.

### Microscopy

Confocal fluorescence imaging was performed using NIS Elements imaging software on a Nikon confocal spinning disk system equipped with an Andor EMCCD camera, a CSU-X1 confocal scanner (Yokogawa), 405, 488, and 561 nm solid-state lasers, and 455/50, 525/26 and 605/70 nm emission filters. Transgenic animals expressing sfGFP_11_ or split-wrmScarlet_11_ were screened using a Leica M165 FC fluorescent stereomicroscope equipped with a Sola SE-V with GFP and mCherry filters.

### Image analysis

Images were analyzed using Fiji. Image manipulations consisted of maximum intensity projections along the axial dimension, rolling ball radius background subtraction, smoothing, and LUT minimum and maximum adjustments. Masks were created by thresholding and setting the pixels under the threshold cutoff to NaN. Plotting of values per pixel was carried out in python 3, using numpy and matplotlib. When performing normalizations for split-sfCherry3 versus split-wrmScarlet, the red channel was divided by the green channel (mNeonGreen::FP_1–10_) because the localization of both fragments is expected to be the same (cytosolic). For normalization of signals where mTagBFP::FP_11_ was targeted to the membrane, the blue channel was used instead of the green channel.

### Mounting worms for microscopy

Pads made of 3% agarose (GeneMate) were dried briefly on Kimwipes (Kimtech) and transferred to microscope slides. Approximately 10 μL of 2 mM levamisole (Sigma) was pipetted onto the center of the agarose pad. Animals were transferred to the levamisole drop, and a coverslip was placed on top before imaging.

### Brood size analysis

Eight single synchronized adults grown at 20°C were transferred to fresh plates every 24 h until cessation of reproduction, and the number of viable progeny produced by each worm was scored.

### Developmental toxicity assay

Ten N2E wild-type animals were microinjected with either *Peft-3::3NLS::mTagBFP2::split-wrmScarlet_11_::T2A::mNeonGreen::split-wrmScarlet_1_*_–_*_10_::fib-1 3’UTR* or *Peft-3::3NLS::mTagBFP2::sfCherry3_11_::T2A::mNeonGreen::sfCherry3_1_*_–_*_10_::fib-1 3’UTR* construct at (20 ng/μL) and were singled. mNeonGreen-positive F_1_ animals were scored and their development was monitored for up to 5 days from egg-laying. The number of fluorescent dead eggs, arrested larvae (*i.e.*, animals never reaching adulthood) or adults were scored for each group.

### Comparison of split-sfCherry3 to split-wrmScarlet in muscle

Ten N2E wild-type animals were microinjected with either *Pmyo-3::mTagBFP2::split-wrmScarlet_11_::T2A::mNeonGreen::split-wrmScarlet_1–10_::fib-1 3’UTR*, or *Pmyo-3::mTagBFP2::sfCherry3_11_::T2A::mNeonGreen::sfCherry3_1_*_–_*_10_::fib-1 3’UTR* constructs were microinjected at (20 ng/μL). F_1_ animals expressing mNeonGreen in muscle were selected for comparison.

### Lifespan assays

NGM plates were supplemented with 5-Fluorouracil (5-FU, Sigma, 15 μM) (Goudeau *et al.* 2011) in order to prevent progeny from hatching and with kanamycin sulfate to prevent bacterial contamination (Sigma, 25 μg/mL). Animals fed with kanamycin-resistant OP50 were scored manually as dead or alive, from their L4 larval stage defined as day 0. A worm was considered alive if it moved spontaneously or, in cases where it wasn’t moving, if it responded to a light touch stimulus with a platinum wire. Animals that crawled off the plates, had eggs that accumulated internally, burrowed or ruptured were censored and included in the analysis until the time of censorship.

### Structure prediction and rendering of split-wrmScarlet

Phyre2 was used to predict the three-dimensional modeling in intensive mode with default parameters (Kelley *et al.* 2015). The 3D model obtained was visualized using PyMOL (v2.2.0).

### Statistical analysis

Differences in fluorescence intensity between groups were compared using unpaired *t*-test with Welch’s correction. Data are presented as means ± SD. Kaplan-Meier estimates of survival curves were calculated using *survival* (v2.38–3) and *rms* (v4.5–0) R packages and differences were tested using log-rank test. The number of animals used in each experiment is indicated in the figure legends.

### Data availability

Strains expressing a single-copy of split-wrmScarlet_1–10_ and/or sfGFP_1–10_ CF4582 (https://cgc.umn.edu/strain/CF4582), CF4587 (https://cgc.umn.edu/strain/CF4587), CF4588 (https://cgc.umn.edu/strain/CF4588), CF4610 (https://cgc.umn.edu/strain/CF4610), DUP223 (https://cgc.umn.edu/strain/DUP223), and DUP237 (https://cgc.umn.edu/strain/DUP237) are available via the *Caenorhabditis* Genetics Center (CGC). The vectors pJG100 carrying *Peft-3::split-wrmScarlet_1_*_–_*_10_::unc-54 3’UTR* (www.addgene.org/138966), pJG103 carrying split-wrmScarlet_11_ x3 tandem repeats (www.addgene.org/158533), yeast plasmids p416-TEF-membrane localization signal-mTagBFP-split-wrmScarlet_11_-TEF terminator (www.addgene.org/158585) and pRS423-GPD-split-wrmScarlet_1–10_-CYC1 terminator (www.addgene.org/158584) are deposited, along with sequences and maps, at Addgene. Other strains and plasmids are available upon request. The authors state that all data necessary for confirming the conclusions presented here are represented fully within the article. A detailed protocol to generate *C. elegans* with sfGFP_11_ and/or split-wrmScarlet_11_ integrants is available at dx.doi.org/10.17504/protocols.io.bamkic4w. Supplementary material is available at figshare: https://doi.org/10.25386/genetics.13635614.

## Results

### Split-wrmScarlet

To engineer split-wrmScarlet, first we introduced a 32 amino acid spacer between the 10th and 11th β-strands of full-length yeast-codon optimized mScarlet, following a strategy described previously (Feng *et al.* 2017). We subjected the spacer-inserted mScarlet sequence to several rounds of semi-random mutagenesis in *E. coli*, generating a version with fluorescence comparable to the full-length mScarlet when expressed in bacteria. However, upon separating the two fragments into two *S. cerevisiae* plasmids to test for complementation, we observed no detectable fluorescence in yeast. We decided to continue with several rounds of selection of new mutant libraries in yeast using FACS, by fusing the small fragment (without the MDELYK C-terminus residues) from our brightest *E. coli* clone to a plasma-membrane-targeted blue FP (mTagBFP), and expressing the large fragment from a high-copy number vector containing a strong promoter. The brightest resulting protein, which we named split-wrmScarlet, contained 10 amino acid substitutions relative to the C-terminal truncated mScarlet (Supplementary Figure S1, A and B). Fluorescence microscopy of yeast containing both plasmids corroborated that split-wrmScarlet showed the expected localization and can reach brightness comparable to that of intact mScarlet in yeast (Supplementary Figure S2, A and B).

### Split-wrmScarlet is threefold brighter than split-sfCherry3 in *C. elegans* muscles

In order to compare split-wrmScarlet to split-sfCherry3, the brightest published red split-FP at the time of the experiment, we combined the FP_1–10_ and FP_11_ fragments into a single plasmid for each fluorophore. Specifically, we generated worm-codon-optimized plasmids encoding three nuclear localization signals (NLS), mTagBFP2, FP_11_, a T2A peptide-bond-skipping sequence, mNeonGreen and the corresponding FP_1–10_, driven by the *eft-3* promoter (Supplementary Figure S3A). Each FP_11_ was linked to mTagBFP2 in order to reduce the risk of proteolysis of the short peptide, and mNeonGreen was linked to FP_1–10_ to monitor its expression, and for normalization purposes. Each construct was injected into wild-type animals and fluorescent progeny were analyzed. Unexpectedly, split-sfCherry3 turned out to be toxic when expressed ubiquitously, whereas 99% of split-wrmScarlet-overexpressing worms became viable adults (Supplementary Figure S3B).

In an attempt to reduce split-sfCherry3-associated toxicity, we modified our construct by using the muscle-specific *myo-3* promoter and removing the NLS sequence (Figure 1B). We did not detect toxicity associated with the expression of these constructs and were able to compare the fluorescence of split-sfCherry3 and split-wrmScarlet in young adults. Red fluorescence emitted from split-wrmScarlet was 2.9-fold higher than that of split-sfCherry3 when normalized to the mNeonGreen signal (Figure 1, B and C and Supplementary Figure S4). We also observed a 60% higher expression level of mTagBFP2 in the split-wrmScarlet-expressing animals (Supplementary Figure S4). It is worth noting that differences in expression levels could influence both brightness and toxicity comparisons. A more controlled way to compare the split FPs at similar expression levels would be to make single-copy genomic insertions of these constructs at a neutral site in the genome.

### Split-wrmScarlet_11_-mediated tagging in all somatic tissues or specifically in muscles

Our protein-tagging approach was analogous to existing split-FP methods developed for human cells (Kamiyama *et al.* 2016, Leonetti *et al.* 2016) and *C. elegans* (Hefel and Smolikove 2019). It requires split-wrmScarlet_1–10_ (*i.e.*, just the large fragment of split-wrmScarlet without the 11th β-strand) to be expressed in the cell or tissue of interest, and the small split-wrmScarlet_11_ fragment to be inserted at an endogenous locus to tag a protein of interest (Figure 1A).

**Figure 2:**
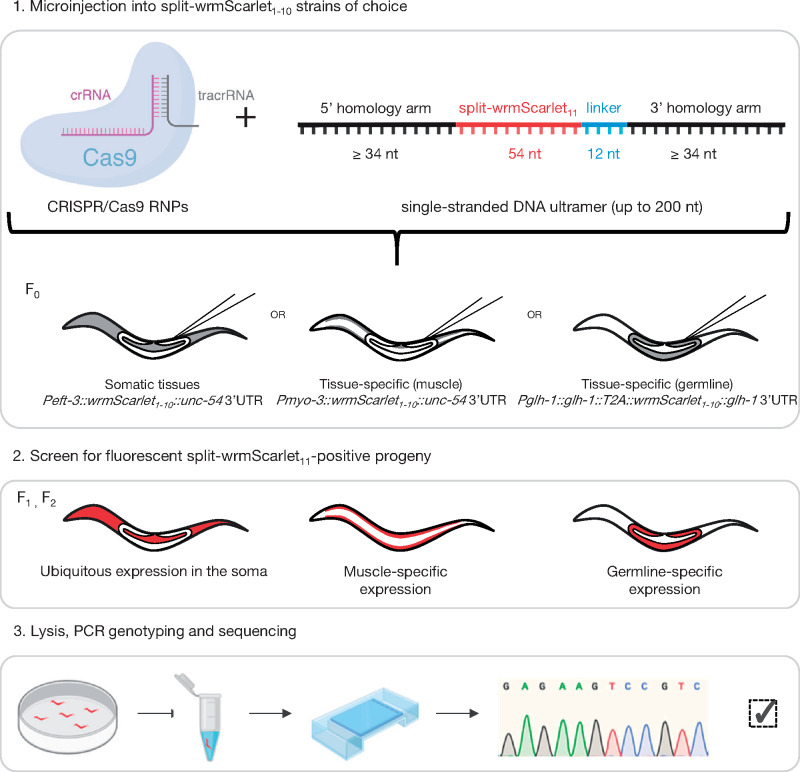
Split-wrmScarlet_11_-mediated tagging. Schematic representation of the split-wrmScarlet tagging workflow to visualize endogenous proteins specifically in muscles, germline, or throughout the soma. Some illustrations were created with BioRender.com.

To build strains expressing single-copy insertions of split-wrmScarlet_1–10_, we first optimized its sequence for *C. elegans* codon usage (Redemann *et al.* 2011) and included three introns (Supplementary Table S1). The strain expressing split-wrmScarlet_1–10_ throughout the soma (driven by the *eft-3* promoter and *unc-54* 3’UTR) was generated by editing the genome of the existing MosSCI line CA1200 (Zhang *et al.* 2015) and replacing the sequence encoding *tir-1::mRuby* with *split-wrmScarlet_1–10_* using CRISPR/Cas9 and hybrid DNA templates (Paix *et al*. 2015; Dokshin *et al.* 2018; Supplementary Table S4). In order to perform tissue-specific labeling, we generated a strain expressing muscle-specific split-wrmScarlet_1–10_ using the SKI-LODGE system in the strain WBM1126 (Silva-García *et al.* 2019; Supplementary Table S4). The expression of split-wrmScarlet_1–10_ in these two lines did not affect the number of viable progeny (Supplementary Figure S5A) nor lifespan (Supplementary Figure S5B and Table S6), suggesting that the expression of split-wrmScarlet_1–10_ had no deleterious effect. To tag a gene of interest with the split-wrmScarlet_11_ fragment, we used microinjection of preassembled Cas9 ribonucleoproteins, because this method enables high-efficiency genome editing in worms (Paix *et al*. 2015). The most efficient insertion of short sequences in *C. elegans* was previously shown to be achieved using ssODNs (Paix *et al*. 2015; Prior *et al.* 2017; Dokshin *et al.* 2018). A great advantage of this strategy is that all of the components required for editing are commercially available or can be synthesized rapidly in the lab (Leonetti *et al.* 2016). Synthetic ssODNs have a typical size limit of 200 nt. The small size of split-wrmScarlet_11_ (18–24 a.a.) is key: 200 nt can encompass split-wrmScarlet_11_ (66–84 nt, including a 4 a.a. linker) flanked by two homology arms >34 nt (up to 67–58 nt) for HDR. In principle, a few days after the somatic and/or muscle-specific split-wrmScarlet_1–10_ strain(s) are microinjected, progeny can be screened for red fluorescence, genotyped and sequenced to check the accuracy of editing (Figure 2; a detailed protocol is available at dx.doi.org/10.17504/protocols.io.bamkic4w). If desired, co-CRISPR strategies such as *dpy-10(cn64)* (Paix *et al*. 2015) or co-injection with pRF4 (Dokshin *et al.* 2018) can be used to screen for correct candidates and to control for microinjection efficacy and payload toxicity.

**Figure 3:**
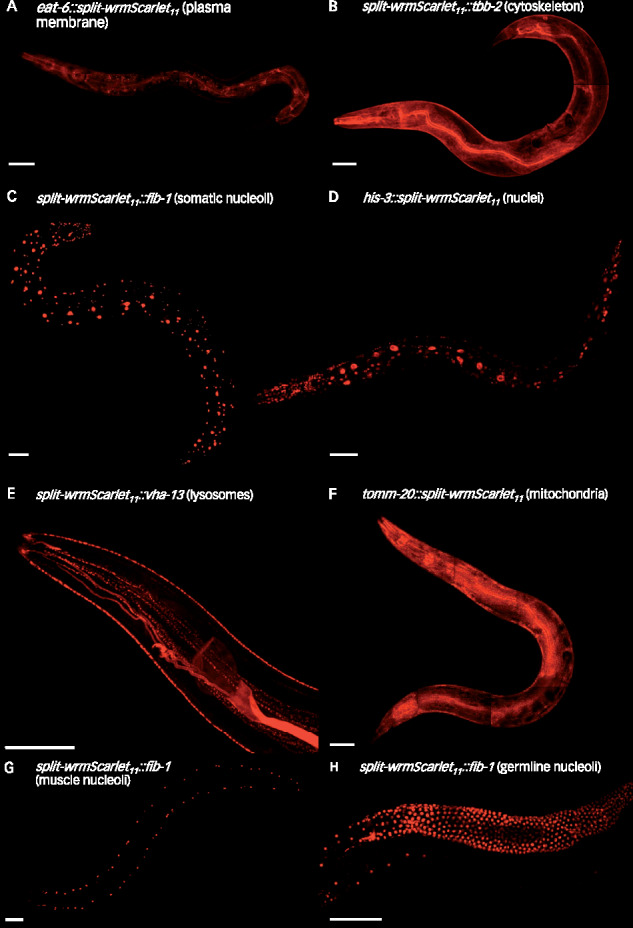
Tissue-specific split-wrmScarlet labeling of proteins with distinct subcellular locations. Endogenous proteins tagged with split-wrmScarlet_11_ in animals expressing split-wrmScarlet_1–10_ in somatic tissues, in muscles or in the germline. (A–F) Confocal images of worms expressing somatic split-wrmScarlet_1–10_ and (A) EAT-6::split-wrmScarlet_11_ (plasma membrane), (B) split-wrmScarlet_11_::TBB-2 (cytoskeleton), (C) split-wrmScarlet_11_::FIB-1 (nucleoli), (D) HIS-3::split-wrmScarlet_11_ (nuclei), (E) split-wrmScarlet_11_::VHA-13 (lysosomes), or (F) TOMM-20::split-wrmScarlet_11_ (mitochondria). (G) Transgenic worm expressing split-wrmScarlet_1–10_ in muscle and split-wrmScarlet_11_::FIB-1. (H) Transgenic worm expressing split-wrmScarlet_1–10_ in the germline and split-wrmScarlet_11_::FIB-1. (A–H) Maximum intensity projections of 3D stacks shown. Scale bars, 50 µm.

To test our approach, we used it to tag six proteins with distinct subcellular localizations. Starting with the somatic split-wrmScarlet_1–10_ parental strain CF4582, we introduced split-wrmScarlet_11_ at the N-terminus of TBB-2, FIB-1 or VHA-13 or at the C-terminus of EAT-6, HIS-3, and TOMM-20 (Supplementary Table S4). These proteins mark the cytoskeleton, nucleoli, lysosomes, plasma membrane, nuclei, and mitochondria, respectively. Importantly, for tagging transmembrane proteins, the split-wrmScarlet_11_ tag was introduced at the terminus exposed to the cytosol. Split-wrmScarlet fluorescence from all six proteins matched their expected subcellular localization in somatic cells (Figure 3, A–F). To test the muscle-specific split-wrmScarlet_1–10 _line CF4610, we tagged the N-terminus of endogenous FIB-1 with split-wrmScarlet_11_ and confirmed the fluorescence from nucleoli in muscle cells (Figure 3G). Together, our results show that split-wrmScarlet enables rapid fluorescent tagging of proteins with disparate cytoplasmic or nuclear locations expressed from their endogenous loci.

The 18 a.a. split-wrmScarlet_11_ sequence used for these experiments ends with two glycines. In mammalian cells, C-terminal gly–gly sequences have been reported to function as degradation signals (Koren *et al.* 2018). Our TOMM-20::split-wrmScarlet_11_ had a spontaneous mutation of the last glycine to a stop codon (Supplementary Table S4), which could be problematic if the protein degradation mechanism, DesCEND (destruction via C-end degrons) operates in *C. elegans*. However, we do not detect differences in protein abundance of HIS-3 versus HIS-3::split-wrmScarlet_11_ by western blots in *C. elegans* (Supplementary Figure S11)*.* We also do not detect differences in protein abundance of mScarlet truncated to end in gly–gly compared to mScarlet ending in MDELYK via fluorescence in yeast. Nonetheless, we recommend using a 24 a.a. split-wrmScarlet_11_ sequence YTVVEQYEKSVARHCTGGMDELYK when labeling proteins at their C-terminus to avoid the possibility that split-wrmScarlet_11_ ending in gly–gly could function as a degron. This modified sequence still fits within the 200 nt ssODN synthesis limit and works at least as well as the 18 a.a. split-wrmScarlet_11_ sequence (Supplementary Figure S6).

### Split-wrmScarlet_11_-mediated tagging in the germline

Our initial attempt to use split-wrmScarlet in the germline failed. We made a single-copy integrated *Psun-1::split-wrmScarlet_1_*_–_*_10_::sun-1* 3’UTR strain via MosSCI, but when we injected a plasmid encoding mNeonGreen::split-wrmScarlet_11_, we observed green fluorescence, but no red fluorescence (Supplementary Figure S7, A and B), suggesting the absence of split-wrmScarlet_1–10_ expression. We suspected germline silencing of the germline-expressed *split-wrmScarlet_1_*_–__*10*_, so we attempted an alternative expression approach by taking advantage of the germline-helicase protein GLH-1, which is highly expressed and germline-specific (Marnik *et al.* 2019). We fused a *T2A::split-wrmScarlet_1_*_–__*10*_ sequence to the C-terminus of the endogenous *glh-1* gene using CRISPR/Cas9. The high expression of GLH-1 yielded high expression of split-wrmScarlet_1–10_, and the T2A separated split-wrmScarlet_1–10_ from GLH-1 (Liu *et al.* 2017). The *glh-1::T2A::split-wrmScarlet_1_*_–__*10*_ strain (https://cgc.umn.edu/strain/DUP237) can be used like our other tissue-specific strains for germline-specific tagging. To demonstrate this, we tagged the N-terminus of endogenous FIB-1 with split-wrmScarlet_11_, and we observed red fluorescence localized to the nucleoli specifically in the germline and embryos, as we hoped (Figure 3H; Supplementary Figure S8, A and C). Finally, we note that the strategy used to express *wrmScarlet_1-10_* or *sGFP2_1-10_* in the germline, by tagging the 3’ end of the endogenous *glh-1* with *T2A::FP_1-10_* with CRISPR/Cas9, could be used to express any other protein of choice.

### Split-wrmScarlet_11_ tandem repeats increase fluorescence

**Figure 4:**
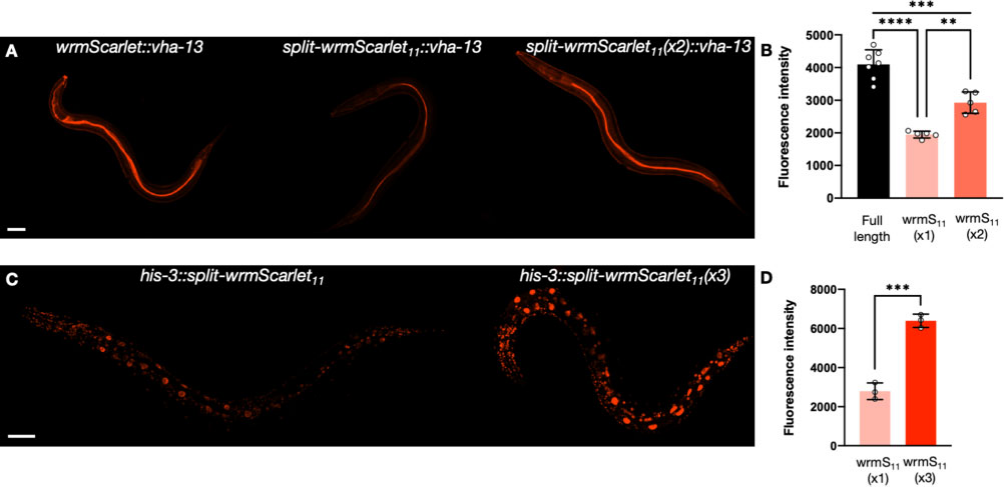
Split-wrmScarlet_11_ tandem repeats increase fluorescence. (A) Images of animals carrying either wrmScarlet, split-wrmScarlet_11_ or two tandem repeats of split-wrmScarlet_11_ inserted at the endogenous VHA-13 N-terminus. (B) Emission intensities of animals carrying wrmScarlet, split-wrmScarlet_11_ or dual split-wrmScarlet_11_ inserted at the VHA-13 N-terminus. Mean ± SD. Circles are individuals. ****P < 0.0001, ***P < 0.001, **P < 0.005. (C) Images of animals carrying either a single split-wrmScarlet_11_ or three tandem repeats of split-wrmScarlet_11_ inserted at the HIS-3 C-terminus. (D) split-wrmScarlet emission intensities from animals carrying a single split-wrmScarlet_11_ or three tandem repeats of split-wrmScarlet_11_ knock-in at the HIS-3 C-terminus. Mean ± SD. Circles are individuals. ***P < 0.001. Images from each comparison were taken under identical instrument conditions using confocal microscopy and are shown using identical brightness and contrast settings. Images shown are from a single confocal plane. Scale bars, 50 µm.

To benchmark the fluorescence intensity of split-wrmScarlet against its full-length counterpart, we first generated endogenously-expressed *wrmScarlet::vha-13* (El Mouridi *et al.* 2017) transgenic animals and compared their fluorescence to *split-wrmScarlet_11_::vha-13* in worms expressing split-wrmScarlet_1–10_ somatically (Figure 4, A and B). At the *vha-13* locus, split-wrmScarlet was about half as bright as a full-length fluorophore (48%), a ratio comparable to that of split-mNeonGreen2 and its full-length counterpart in human cells (Feng *et al.* 2017).

Since visualizing endogenous proteins of low abundance can be challenging, it is key to address this limitation. Increasing the number of FP_11_ domains tagged to an endogenous protein multiplies the number of the corresponding FP_1–10_s recruited, increasing the overall fluorescent signal in human cells (Leonetti *et al.* 2016) and in *C. elegans* (He *et al.* 2019; Hefel and Smolikove 2019). To demonstrate that split-wrmScarlet fluorescence is enhanced by split-wrmScarlet_11_ tandem repeats, we introduced two split-wrmScarlet_11_ domains at the N-terminus of VHA-13 and three split-wrmScarlet_11_ domains at the C-terminus of HIS-3 in animals expressing somatic split-wrmScarlet_1–10_. Compared to animals carrying a single split-wrmScarlet_11_ at the identical locus, carrying two split-wrmScarlet_11_s increased overall fluorescence by 1.5-fold, while carrying three increased it by 2.3-fold (Figure 4, C and D). Note that our three-split-wrmScarlet_11_ tandem sequence exceeds the 200 nt ssODN synthesis limit, so we used dsDNA donor templates for these constructions (Supplementary Table S4).

### sfGFP_11_-mediated tagging in somatic cells

**Figure 5:**
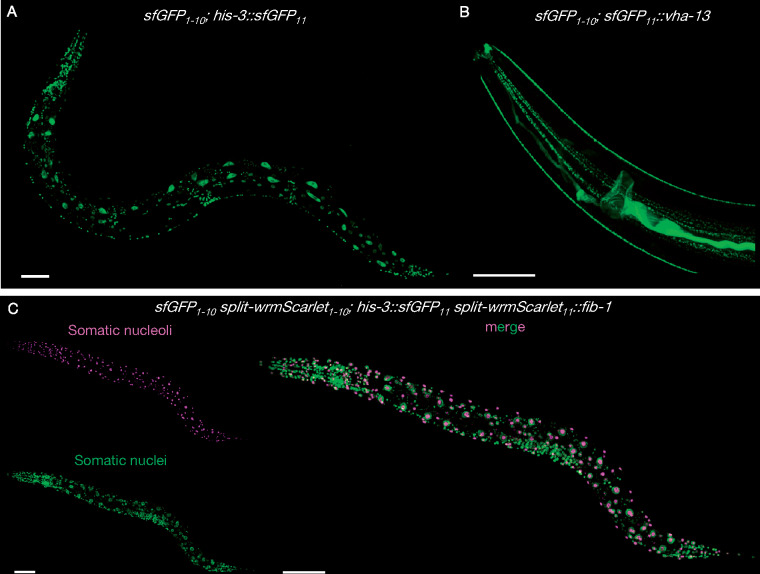
Split-sfGFP and split-wrmScarlet dual-color protein labeling. Images of animals stably expressing sfGFP_1–10_ in somatic tissues (A) CF4592 *muIs253[Peft-3::sfGFP_1–10_::unc-54 3'UTR Cbr-unc-119(+)] II; unc-119(ed3) III; his-3(muIs255[his-3::sfGFP_11_]) V* or (B) CF4589 *muIs253[Peft-3::sfGFP_1–10_::unc-54 3'UTR Cbr-unc-119(+)] II; unc-119(ed3) III; vha-13(muIs268[sfGFP_11_::vha-13]) V*. (C) Dual color protein labeling with split-wrmScarlet and split-sfGFP in somatic cells. Composite display of red and green channels of animals expressing split-wrmScarlet_1–10_ and sfGFP_1–10_ in somatic tissues, HIS-3::sfGFP_11_ and split-wrmScarlet_11_::FIB-1; CF4602 *muIs253[Peft-3::sfGFP_1–10_::unc-54 3'UTR Cbr-unc-119(+)] muIs252[Peft-3::split-wrmScarlet_1–10_::unc-54 3'UTR Cbr-unc-119(+)] II; unc-119(ed3) III; fib-1(muIs254[split-wrmScarlet_11_::fib-1]) his-3(muIs255[his-3::sfGFP_11_]) V*. Maximum intensity projections of 3D stacks shown. Scale bars, 50 µm.

Split-sfGFP has been used successfully in worms before (Noma *et al.* 2017; He *et al.* 2019; Hefel and Smolikove 2019). However, there is still a need for a strain that ubiquitously expresses sfGFP_1–10_ in the soma from an integrated single-copy insertion in order to avoid heterogeneous expression and time-consuming manual maintenance. To build this strain, we codon-optimized the original *sfGFP_1–10_* sequence for *C. elegans* and included one intron (Cabantous *et al.* 2005; Redemann *et al.* 2011; Supplementary Table S1). We initially generated a strain expressing sfGFP_1–10_ driven by the *let-858* promoter and *unc-54* 3’UTR using MosSCI, but later replaced the *let-858* promoter with the *eft-3* promoter using CRISPR/Cas9 and hybrid DNA donor template because we observed that *Peft-3* resulted in significantly higher levels of gene expression (Paix *et al*. 2015; Dokshin *et al.* 2018; Supplementary Table S4). To validate this strain, we inserted sfGFP_11_ at the N-terminus of lysosomal VHA-13 or at the C-terminus of nuclear-localized HIS-3 (Figure 5, A and B). Both strains yielded relatively bright signals in accordance with their predicted subcellular localization. We generated eGFP::VHA-13 transgenic animals and compared their fluorescence to sfGFP_11_::VHA-13 in worms expressing sfGFP_1–10_ somatically (Supplementary Figure S9, A and B). At the *vha-13* locus, split-sfGFP was about a third as bright as a full-length eGFP. It is worth noting that this comparison is not perfect, in part due to the presence of the six superfolder mutations S30R, Y39N, N105T, Y145F, I171V, and A206V in sfGFP but not in eGFP.

### sGFP2_11_-mediated tagging in the germline

We also generated a germline-specific sGFP2_1–10_ strain using a similar strategy (Supplementary Figure S8B, Supplementary Table S1). Split-sGFP2 is a split-superfolder GFP variant optimized for brightness and photostability (Köker *et al.* 2018). To test this germline-specific line DUP223 (https://cgc.umn.edu/strain/DUP223), we tagged the C-terminus of endogenous PGL-1 with sGFP2_11_ and confirmed the green fluorescence from P-granules (Supplementary Figure S8B, lower panel).

### Dual color protein labeling with split-wrmScarlet and split-sfGFP

Finally, to allow two-color imaging in the soma, we crossed the strains CF4592 *Peft-3::sfGFP_1_*_–_*_10_; his-3::sfGFP_11_* and CF4601 *Peft-3::split-wrmScarlet_1_*_–_*_10_; split-wrmScarlet_11_::fib-1*. This cross resulted in the line CF4588 *Peft-3::sfGFP_1_*_–_*_10_ Peft-3::split-wrmScarlet_1_*_–__*10*_ (https://cgc.umn.edu/strain/CF4588) as well as the dually labeled strain CF4602 *Peft-3::sfGFP_1_*_–_*_10_ Peft-3::split-wrmScarlet_1_*_–_*_10_; split-wrmScarlet_11_::fib-1 his-3::sfGFP_11_* (Figure 5C). The fluorescent signals from both split-FPs appeared in their respective subcellular compartments, suggesting the two systems are compatible. We note an additional advantage of the strain CF4588: the loci of *split-wrmScarlet_1_*_–__*10*_ and *sfGFP_1–10_* are genetically linked (only 0.96 cM apart), which facilitates outcrossing when needed. In addition, all our parental *C. elegans* lines expressing *split-wrmScarlet_1_*_–__*10*_ and *sfGFP_1_*_–__*10*_ are viable homozygotes, so the strains do not require special maintenance.

### The current split-wrmScarlet is not detectable in mammalian cells

We failed to detect split-wrmScarlet in mammalian cells, despite our efforts to rescue its fluorescence by screening a mammalian-codon-optimized split-wrmScarlet_11_ single/double mutant library in HEK293T cells (Supplementary Figure S10, A and B, and Supplementary text).

## Discussion

In this study, we describe several new tools for rapid CRISPR-mediated labeling of endogenously expressed proteins using split fluorophores. While these tools are powerful and relatively easy to implement, several considerations should be taken into account when using this method.

First, detection of a given protein labeled with an FP_11_ can only occur in a cellular compartment where the corresponding FP_1–10_ is present. Proteins tagged with split-wrmScarlet_11_ or sfGFP_11_ generated in this work were either exposed to the cytosol or nucleoplasm (nuclei or nucleoli), where split-wrmScarlet_1–10_ and/or sfGFP_1–10_ were present. For proteins or epitopes located within the lumen of organelles, such as mitochondria or the endoplasmic reticulum, one might need to generate and validate *C. elegans* lines expressing split-wrmScarlet_1–10_ or sfGFP_1–10_ containing a mitochondrial localization sequence or ER signal peptide and retention signals, respectively. These approaches have been used successfully in mammalian cells with split-sfGFP when tagging ER-resident polypeptides (Kamiyama *et al.* 2016) and with split-sfCherry2 to detect proteins present in the mitochondrial matrix (Ramadani-Muja *et al.* 2019).

Second, when labeling proteins with split-wrmScarlet at the C-terminus, we recommend using the 24 a.a. split-wrmScarlet_11_ sequence YTVVEQYEKSVARHCTGGMDELYK. As described in the Results section, our 18 a.a. split-wrmScarlet_11_ fragment ends in gly–gly, which has been shown to be a degradation signal in mammalian cells. We cannot exclude the possibility that ending in gly–gly can be detrimental in *C. elegans*. The 24 a.a. split-wrmScarlet_11_ still fits within a 200 nt ssODN donor template with a 12 nt linker and up to 58 nt homology arms and is at least as bright as the 18 a.a. split-wrmScarlet_11_ (Supplementary Figure S6).

Third, as for any other protein tag, it is important to select, when possible, a site that is unlikely to interfere with protein folding, function or localization (Snapp 2005; Nance and Frøkjær-Jensen 2019). For example, N-termini of membrane- and organelle-resident proteins often contain signal peptides or localization signals, and C-termini may contain degron sequences that regulate protein turnover. Interestingly, there are examples of proteins that become toxic when tagged with a full-length GFP, but tolerate labeling with a split fluorescent protein. For example, SYP-4 was reported to be mostly functional when endogenously tagged with sfGFP_11_ in a strain expressing sfGFP_1–10_ specifically in the germline, but not functional when labeled with full-length GFP (Hefel and Smolikove 2019).

Fourth, for proteins of interest present at low levels, we provided an alternative protocol to insert an additional two or three split-wrmScarlet_11_ fragments, which increases the overall fluorescence substantially. However, the number of split-wrmScarlet_11_ fragments could likely be increased further, to at least seven tandem repeats, based on approaches used successfully with split-sfGFP in human cells (Feng *et al.* 2017) and *C. elegans* (Noma *et al.* 2017; He *et al.* 2019; Hefel and Smolikove 2019).

Fifth, we would like to emphasize differences between our technique and the bimolecular fluorescence-complementation (BiFC) assay. When used together, high-affinity green and red split fluorescent proteins can provide information on co-localization, but unlike BiFC split proteins (Hu *et al.* 2002), they are not intended to assess protein-protein interactions directly. This is because BiFC split proteins require finely tuned weak affinities that do not disrupt the underlying interaction being studied. In our approach, only the split-wrmScarlet_11_ fragment is attached to a protein of interest, the split-wrmScarlet_1–10_ fragment is expressed in excess and unattached.

Finally, we would like to note that despite being three times brighter than the latest split-sfCherry3 in worms, our current split-wrmScarlet was not visible in the mammalian cell line we examined (Supplementary Figure S10). Its ability to fluoresce is not restricted to worms, because it can reach wild-type levels of brightness in yeast. We do not know the basis for this discrepancy. It is possible that the concentration of the split-wrmScarlet_1–10_ fragment in mammalian cells is too low to drive complementation with split-wrmScarlet_11_. This could potentially be overcome by further mutagenizing split-wrmScarlet and screening for fluorescence at low expression levels in mammalian cells.

In conclusion, we believe our system can substantially increase the speed, efficiency, and ease of *in vivo* microscopy studies in *C. elegans*. We expect it to facilitate two-color and co-localization experiments and to find wide use in the worm community. We believe that these strains could facilitate novel or large-scale experiments, such as efforts to tag the entire genome of *C. elegans*.

## Funding

J.G., J.P., A.G.Y., C.K. and M.I. are supported by Calico Life Sciences L.L.C, M.D.L. and L.S. by the Chan Zuckerberg Biohub, and C.S.S. and D.L.U. by NIH-NIGMS (R01 GM113933) with use of equipment supported by NIH-NIGMS (P20 GM103423).

## Conflicts of interest

None declared.
